# A Hybrid Computational Method for the Discovery of Novel Reproduction-Related Genes

**DOI:** 10.1371/journal.pone.0117090

**Published:** 2015-03-13

**Authors:** Lei Chen, Chen Chu, Xiangyin Kong, Guohua Huang, Tao Huang, Yu-Dong Cai

**Affiliations:** 1 Institute of Systems Biology, Shanghai University, Shanghai, 200444, People’s Republic of China; 2 College of Information Engineering, Shanghai Maritime University, Shanghai, 201306, People’s Republic of China; 3 State Key Laboratory of Molecular Biology, Shanghai Key Laboratory of Molecular Andrology, Institute of Biochemistry and Cell Biology, Shanghai Institutes for Biological Sciences, Chinese Academy of Sciences, Shanghai, 200031, People’s Republic of China; 4 Institute of Health Sciences, Shanghai Institutes for Biological Sciences, Chinese Academy of Sciences, Shanghai, 200025, People’s Republic of China; Semmelweis University, HUNGARY

## Abstract

Uncovering the molecular mechanisms underlying reproduction is of great importance to infertility treatment and to the generation of healthy offspring. In this study, we discovered novel reproduction-related genes with a hybrid computational method, integrating three different types of method, which offered new clues for further reproduction research. This method was first executed on a weighted graph, constructed based on known protein-protein interactions, to search the shortest paths connecting any two known reproduction-related genes. Genes occurring in these paths were deemed to have a special relationship with reproduction. These newly discovered genes were filtered with a randomization test. Then, the remaining genes were further selected according to their associations with known reproduction-related genes measured by protein-protein interaction score and alignment score obtained by BLAST. The in-depth analysis of the high confidence novel reproduction genes revealed hidden mechanisms of reproduction and provided guidelines for further experimental validations.

## Introduction

All living creatures generate healthy offspring and maintain population growth through reproduction. In mammals, this fundamental and complex process includes the development of male and female germ cells [1,2], fertilization and embryonic development [3]. Impairment in any of these stages can lead to severe consequences such as infertility, miscarriage and fetal defects. Among mammalian species, humans are more susceptible to reproductive problems. It has been reported that infertility affects approximately 15% of couples [4], and this percentage is increasing. Over the past few decades, mounting evidence has indicated that human fertility and reproduction may be jeopardized by genetic abnormalities [5,6], environmental chemicals [7,8], unhealthy diets and lifestyles [9–11]; however, the underlying molecular mechanisms are still largely unknown. Therefore, it is important to identify reproduction-related genes and pathways that may be used as biomarkers for early diagnosis and treatment.

With advancements in reproductive biology research, a number of reproduction-related genes have been identified and characterized. Their functions are enriched in different reproductive process stages, including gonad development [12,13], germ cell development [14], meiosis [15,16], sperm-egg binding [17,18] and embryo implantation and development [19]. For example, the nanos proteins function in primordial germ cell (PGC) migration into the gonads [14], the TDRP and TNP proteins are involved in spermatogenesis [20,21], and ZP family proteins facilitate the sperm acrosomal reaction and sperm-egg binding [18]. Additionally, several important pathways have proven to be directly involved in reproduction. The Wnt signaling pathway plays a crucial role in gonad development by patterning the sex-specific vasculature and regulating steroidogenic cell recruitment [12]. Not only have these studies promoted the understanding of human reproduction mechanisms but the resulting data have also served as useful resources for deducing new reproductive-related genes and predicting their functions [22,23].

One possible strategy for elucidating the molecular mechanisms underlying the entire reproductive process is to identify all reproduction-related genes and to test their biological roles *in vitro* and *in vivo*. However, such an approach is challenging because the research space, *i*.*e*., the number of human genes, is large, and confirming a reproduction-related gene is temporally and financially intensive.

There are already several network based disease gene identification methods. The basis of most methods is Guilt-by-association [24]. They assumed that the genes are similar with their neighbors. Therefore, the neighbors of the seed disease genes are very likely to be disease genes as well. In general, it is true due to modularized nature of network [25]. But when the seed disease genes are incomplete and scattered overall the whole network, the performance of such method will be poor.

Many other methods are based on Random Walk with Restart (RWR) [26–31]. RWR simulates a walker who randomly walks on the network. It starts from the seed disease genes and moves to its randomly chosen neighbors at each step [28]. After many steps, the probability of the walker walks to each node on the network will be steady. Based on these probabilities, the novel candidate disease genes can be identified.

Different variations of Guilt-by-association and RWR were developed for different research topics, such as neighbor counting [32], RWOAG (https://r-forge.r-project.org/R/?group_id=1126) developed by Kohler *et al*. [28], HumanNet developed by Lee *et al*. [33].

Shortest path based methods have less applications, but in yeast longevity study, it has been proven to be useful for identifying the genetic determinants [34]. And for disease gene identification, there are several successful applications based on shortest path [35–37].

To discover novel reproduction-related genes, we compared these methods and developed a hybrid computational method which integrates the network topology, sequence similarity and protein interaction confidence score. The biological significance of the identified novel reproduction genes were evaluated by enrichment analysis and manually literature survey. Many of the reproduction gene candidates looked very promising.

## Materials and Methods

### 2.1 Materials

The known 115 human reproduction-related genes with experimental evidence in the Gene Ontology database (GO:0000003) were downloaded from the following website: http://amigo.geneontology.org/amigo/term/GO:0000003 (May 10, 2014) [38]. These 115 genes are listed in the S1 Information.

According to the methods in [35–37], to conduct our assessment, we also required data from a protein-protein interaction (PPI) network. We downloaded the file (protein.links.detailed.v9.1.txt.gz) containing the PPI information from STRING (Search Tool for the Retrieval of Interacting Genes/Proteins) (http://www.string-db.org/) [39], a large database containing known and predicted protein interactions. These protein interactions are derived from the following sources: (I) genomic context, (II) high-throughput experiments, (III) (conserved) co-expression and (IV) previous knowledge. Thus, these interactions include physical and functional associations of proteins, which therefore widely measure the relationship between proteins and are different from experimentally determined PPIs provided in some other databases, such as DIP (Database of Interaction Proteins) database [40], BioGRID [41], *etc*. This kind of PPIs has been applied to investigate some protein-related problems [35–37,42–44]. We extracted 1,640,707 PPIs of human from the obtained file, where 86,854 are validated by solid experiments. Each obtained PPI contains two proteins (represented by ensembl IDs) and eight types of score entitled by ‘neighborhood’, ‘fusion’, ‘cooccurence’, ‘coexpression’, ‘experimental’, ‘database’, ‘textmining’, and ‘combined_score’. Since the last type score (with a range of 150–999) integrated the information of other seven types of score, it was used in this study to measure the interaction strength between two proteins. For convenience, it was termed as confidence score and denoted by *S*(*p*_1_, *p*_2_) for a certain PPI containing proteins *p*_1_ and *p*_2_. And proteins *p*_1_ and *p*_2_ were deemed to be interacting proteins if the interaction between them was a member of 1,640,707 PPIs.

### 2.2 Graph-based method to select candidate genes and further selection

Some studies have shown that two interacting proteins are more likely to share similar functions than those that do not interact with each other [45,46]. The interacting proteins of the reproduction-related genes may share some reproductive functions. The interaction confidence scores should also be considered, *i*.*e*., proteins that can interact with known reproduction-related genes with high confidence scores are more likely to possess reproduction-related functions. To test this hypothesis, a weighted graph *G* = (*V*, *E*) was built according to the information of PPIs as follows: *V* consisted of all human proteins occurring in 1,640,707 PPIs and two nodes were adjacent if and only if the corresponding proteins can interact with each other, *i*.*e*., they were interacting proteins. As the range of confidence score is between 150 and 999, a weight was assigned to each edge *e* = (*n*_1_, *n*_2_) by *w*(*e*) = 1000-*S*(*p*_1_, *p*_2_), where *p*_1_ and *p*_2_ are corresponding proteins of nodes *n*_1_ and *n*_2_, respectively. The graph-based method was fully based on this weighted graph *G*. Please refer to our previous studies for detailed information on this method [35–37]. A brief procedure of the method follows:

- **Step 1.** Apply Dijkstra’s algorithm [47] to search all shortest paths in *G* such that their endpoints were known reproduction-related genes.
- **Step 2.** For each node *n* in *G*, count the number of paths obtained in Step 1 which contained *n* as an inner node. This value was termed as “betweenness” in this study. In fact, the betweenness indicates the direct and indirect influences of proteins at distant network [48]. Here, it suggested the direct and indirect association with reproduction-related genes.
- **Step 3.** Select nodes, *i*.*e*., corresponding genes, with betweenness larger than zero as shortest path genes.
- **Step 4.** Randomly produce 500 gene/node sets in *G*. Each produced set had the same size of the set consisting of known reproduction-related genes but was different from it.
- **Step 5.** For each gene set, use the Dijkstra’s algorithm to search all shortest paths in *G* connecting any pair of genes in the set and calculate the betweenness for each shortest path gene obtained in Step 3 based on these shortest paths (betweenness of some shortest path genes may be zero).
- **Step 6.** For each shortest path gene, count the number of gene sets on which the betweenness was larger than that on the set consisting of known reproduction-related genes, thereby calculating the permutation FDR (False Discovery Rate) defined as this value divided by 500.
- **Step 7.** Select shortest path genes whose permutation FDRs were smaller than 0.05 as candidate genes.

### 2.3 Random walk with restart

Random Walk with Restart (RWR) [26–31,49] simulates a random walker starting from m known reproduction genes on the network and moves to its randomly chosen interaction neighbors at each step [28]. In each step, the state probabilities *P*_*t* + 1_ at time *t* + 1 is

$$P_{t+1}=\left(1-r\right)A^{'}P_{t}+r$$ (1)

where *P*_*t* + 1_ is state probabilities at time *t*, *r* is the restart probability, 0.7 as suggested by previous literatures [28], *A* is the column-wise normalized adjacency matrix of the protein interaction network, *P*_0_ is the initial state probabilities which is a column vector with 1/ *m* for the *m* known reproduction genes and to 0 for other genes on the protein interaction network.

This process is repeated until the difference between two states is smaller than 1e-6, as suggested by previous literatures [28]. At last, each protein on the network will be assigned with a probability of being novel reproduction gene.

R package RWOAG [28] from https://r-forge.r-project.org/R/?group_id=1126 was used to apply RWR.

### 2.4 Similarity-based method to select candidate genes

Using the properties of protein sequences to study various protein-related problems is a classic approach. Blast (basic local alignment search tool), proposed by Altschul *et al*. [50], is a well-known tool that can search local similarity between two protein sequences. For two proteins *p*_1_ and *p*_2_, the alignment score of their sequences, denoted by *S*_*b*_(*p*_1_, *p*_2_), can be obtained by BLAST.

It is known that two proteins with high alignment score have similar structures, thereby sharing similar functions. Thus, using the alignment scores to identify novel reproduction-related genes is an alternative method. For formulation, let *S* be a training gene set consisting of two parts: *S*_*R*_ and *S*_*NR*_, where *S*_*R*_ comprised known reproduction-related genes, *S*_*NR*_ was composed of other genes that had not been validated to be reproduction-related genes. For a query gene *p*, we can calculate two values as follows:

$$v_{R}^{b}\left(p\right)=\text{max}\left\{S_{b}\left(p,p^{'}\right)|p^{'}\in S_{R}\right\}$$ (2)

$$v_{NR}^{b}\left(p\right)=\text{max}\left\{S_{b}\left(p,p^{'}\right)|p^{'}\in S_{NR}\right\}$$ (3)

It is easy to observe that $v_{R}^{b}\left(p\right)$ measures the structure associations between *p* and genes in *S*_*R*_, whereas $v_{NR}^{b}\left(p\right)$ measures the structure associations between *p* and genes in *S*_*NR*_. Specifically, the high values of $v_{R}^{b}\left(p\right)$ and $v_{NR}^{b}\left(p\right)$ indicate strong structure associations. In view of this, if $v_{R}^{b}\left(p\right)>v_{NR}^{b}\left(p\right)$, then *p* is identified to be a candidate gene for reproduction.

### 2.5 Interaction-based method to select candidate genes

As mentioned in Section 2.2, interacting proteins of proteins encoded by reproduction-related gene may share some reproductive functions, thereby inferring that genes encoding these proteins may be reproduction-related gene. Thus, we can directly use the information concerning PPIs to identify possible reproduction-related genes. We still used the notations defined in Section 2.4. For a query gene *p*, we can calculate two values as follows:

$$v_{R}^{i}\left(p\right)=\text{max}\left\{S\left(p,p^{'}\right)|p^{'}\in S_{R}\right\}$$ (4)

$$v_{NR}^{i}\left(p\right)=\text{max}\left\{S\left(p,p^{'}\right)|p^{'}\in S_{NR}\right\}$$ (5)

With the similar argument in Section 2.4, we can identify *p* as a candidate gene for reproduction if $v_{R}^{i}\left(p\right)>v_{NR}^{i}\left(p\right)$.

### 2.6 Hybrid method to select candidate genes

The hybrid method partly combined the methods described in Section 2.2, 2.4 and 2.5. Since graph-based method is similar with RWR and graph-based method has better performance than RWR (see Section 3.1), we chose graph-based method to represent network method and then integrated with similarity-based method and interaction-based method.

The purpose of this study is to discover new candidate reproduction-related genes, it is difficult to completely integrate the similarity-based method and interaction-based method because the set *S*_*NR*_ is difficult to be well-defined. In view of this, we only used **Eq. 2** and **Eq. 4** and set thresholds to select candidate genes. The detailed procedures were as follows:

- The graph-based method was first applied to identify candidate genes for reproduction.
- For each obtained candidate gene *p* in (I), calculate $v_{R}^{b}\left(p\right)$ in the similarity-based method by **Eq. 2**, where *S*_*R*_ consisted of all reproduction-related genes. Then, exclude the candidate genes with $v_{R}^{b}\left(p\right)$ less than 90.
- For each remaining candidate gene *p* in (II), calculate $v_{R}^{i}\left(p\right)$ in the interaction-based method by **Eq. 4**, where *S*_*R*_ consisted of all reproduction-related genes. Then, exclude the candidate genes with $v_{R}^{i}\left(p\right)$ less than 900.

**Fig 1:**
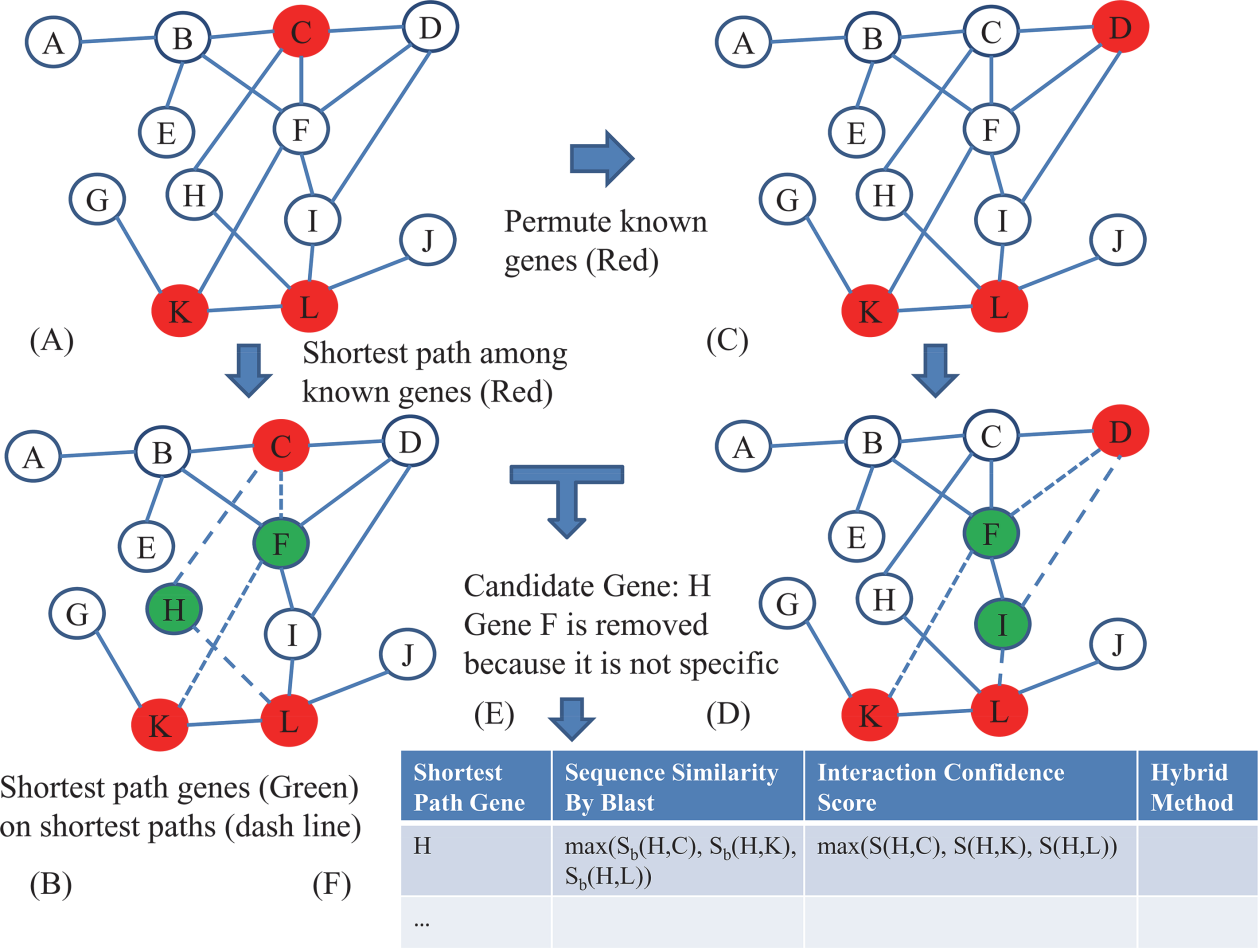
The workflow of hybrid method for novel reproduction gene identification. (A-E) were the steps of graph-based method, (F) was to filter candidates of the graph based method with similarity-based method and interaction-based method. (A) The known reproduction genes (red nodes) were mapped onto network. (B) The shortest path genes (green nodes) on shortest paths (dash line) were identified. (C) The known reproduction genes were permuted. (D) The shortest path genes on the shortest path between permuted reproduction genes were identified. (E) The actual betweenness of shortest path genes were compared with permuted betweenness and the genes that were not specific to reproduction were removed. (F) The candidates of the graph based method were further filtered by checking alignment score and interaction confidence score with known reproduction genes and novel candidate reproduction genes were selected if they were selected by graph-based method, similarity-based method and interaction-based method.

The workflow of the hybrid method was shown in **Fig. 1**.

## Results and Discussions

### 3.1 Comparison of the four methods

This section gave the performance of the four methods described in Section 2.2–2.5 evaluated by the jackknife test, *i*.*e*., one reproduction-related gene was singled out to check whether it can be identified by the rest reproduction-related genes.

To compare the performance of the four methods in a fair circumstance, proteins occurring in PPIs were all considered. 129 shortest path genes with FDR smaller than 0.05 were discovered by graph-based method (see Section 3.2). To make a fair comparison, we considered a gene to be a candidate if its probability of being novel reproduction genes ranks on the top 129^th^ in RWR. For similarity-based and interaction-based methods, the criteria were described in Section 2.4 and 2.5. As a result, the identified reproduction-related genes are listed in **Table 1**, from which we can observe that fourteen, eleven, thirteen and eight reproduction-related genes were identified by graph-based method, RWR method, similarity-based method and interaction-based method, respectively. It is clear that the graph-based method gave the best performance, followed by similarity-based method, RWR method and interaction-based method. Since RWR method and graph-based method were both network method and graph-based method had better performance than RWR, we chose graph-based method over RWR. And we arranged the graph-based method as the first choice in the hybrid method, the similarity-based method as the second choice and the interaction-based method as the last choice. The graph-based method is more likely to find global long distance candidate genes while the similarity-based method and interaction-based method are exploring the local candidates. Therefore, the graph-based candidates may cross several pathways and are scattered overall the network. They may be not significantly enriched onto single pathway, but may reveal novel mechanisms in complex biological systems, such as cross-talks and synergy effects. The false positive rates of similarity-based candidates and interaction-based candidates could be lower since they only explore limited number of local genes. Integrating these methods will balance the novelty and reliability of discovered candidate genes.

**Table 1:** Reproduction-related genes that can be identified by four methods.

Graph-based method		RWR method		Similarity-based method		Interaction-based method	
Ensembl ID	Gene Symbol	Ensembl ID	Gene Symbol	Ensembl ID	Gene Symbol	Ensembl ID	Gene Symbol
ENSP00000009180	CD9	ENSP00000221496	AMH	ENSP00000219593	ZP2	ENSP00000220772	SFRP1
ENSP00000220772	SFRP1	ENSP00000245479	SOX9	ENSP00000238682	TGFB3	ENSP00000228280	KITLG
ENSP00000221496	AMH	ENSP00000288135	KIT	ENSP00000269216	GATA6	ENSP00000241256	GHSR
ENSP00000257963	ACVR1B	ENSP00000297261	SHH	ENSP00000273739	SLIT2	ENSP00000266126	EIF2B2
ENSP00000267430	FANCM	ENSP00000321797	FGF8	ENSP00000290167	WNT4	ENSP00000328169	JAG2
ENSP00000273739	SLIT2	ENSP00000331327	WT1	ENSP00000302951	STRA13	ENSP00000331327	WT1
ENSP00000273783	EIF2B5	ENSP00000333188	FOXL2	ENSP00000332164	SLIT3	ENSP00000369927	AKR1C3
ENSP00000276571	CRH	ENSP00000334458	GATA4	ENSP00000334458	GATA4	ENSP00000380702	MYCBP
ENSP00000288135	KIT	ENSP00000372547	SRY	ENSP00000355896	TGFB2		
ENSP00000290167	WNT4	ENSP00000378326	ZP3	ENSP00000366396	XRN2		
ENSP00000335074	GHRL	ENSP00000384708	FSHR	ENSP00000367494	SPIRE2		
ENSP00000354720	SMC3			ENSP00000372547	SRY		
ENSP00000362690	NR5A1			ENSP00000387266	SPIRE1		
ENSP00000417164	ROBO2						

### 3.2 Candidate genes obtained by the hybrid method

According to the procedures of the hybrid method, the graph-based method was first applied to discover candidate genes for reproduction. By the graph-based method, of the 115 known reproduction-related genes, the shortest paths connecting any two were searched in *G*. The betweenness of each node was calculated, thereby obtaining 406 shortest path genes, which are listed in the S2 Information. According to **Steps 4–6** of the method, the permutation FDR was calculated for each shortest path gene, which is also listed in the S2 Information. The purpose of this procedure is to exclude some genes with both high betweenness and permutation FDRs. If a certain candidate gene can always receive high betweenness for randomly produced gene sets, *i*.*e*., this gene always have strong direct and indirect associations with randomly selected genes, resulting in high permutation FDR, it cannot be deemed to be related to reproduction even if its betweenness was very high. In view of this, this kind of genes should be excluded. By setting the threshold of permutation FDR to 0.05, 129 candidate genes whose permutation FDRs were smaller than 0.05 were obtained, which are listed in the S3 Information. These genes would be further filtered by the following procedures of the hybrid method.

By the second step of hybrid method, for each candidate gene *p*, $v_{R}^{b}\left(p\right)$ was computed according to **Eq. 2**. These values for 129 candidate genes obtained by the graph-based method are listed in S3 Information. Clearly, by setting 90 as a threshold, 27 candidate genes remained, which are listed in S4 Information.

For the remaining 27 candidate genes, the third step of hybrid method was finally used to make selection. The value $v_{R}^{i}\left(p\right)$ was calculated by **Eq. 4** for each candidate gene *p*. Similarly, we set a threshold of 900 to filter these candidate genes, resulting in 21 candidate genes. These genes were deemed to be significant for reproduction and were analyzed for their likelihood to be novel reproduction-related genes in the following sections. The detailed information of these 21 candidate genes are listed in **Table 2**.

**Table 2:** Detailed information of 21 candidate genes obtained by hybrid method.

Ensembl ID	Gene symbol	Betweenness	Permutation FDR	Maximum interaction score to reproduction-related gene (most closely reproduction-related gene)	Maximum alignment score to reproduction-related gene (most closely reproduction-related gene)
ENSP00000364133	TGFBR1	217	0.006	999 (TGFB3)	682 (ACVR1B)
ENSP00000380280	FGFR1	437	0	999 (FGF8)	239 (KIT)
ENSP00000351905	TGFBR2	1	0.028	999 (TGFB3)	231 (ACVR1B)
ENSP00000241416	ACVR2A	266	0	998 (INHBA)	233 (ACVR1C)
ENSP00000266058	SLIT1	212	0	997 (ROBO2)	1995 (SLIT2)
ENSP00000266646	INHBE	199	0	997 (ACVR1B)	149 (INHBA)
ENSP00000309913	TBX5	2	0.034	996 (GATA4)	244 (TBX3)
ENSP00000263640	ACVR1	136	0.002	994 (AMH)	439 (ACVR1C)
ENSP00000250448	FOXA1	5	0.034	993 (SHH)	120 (FOXF2)
ENSP00000245451	BMP4	172	0.012	993 (SHH)	105 (NODAL)
ENSP00000168712	FGF4	107	0.004	992 (SHH)	104 (FGF9)
ENSP00000254227	NR0B2	232	0.018	989 (NR5A1)	129 (NR0B1)
ENSP00000363708	BMPR2	127	0.004	981 (GDF9)	194 (ACVR1B)
ENSP00000364709	F10	107	0.008	980 (SERPINA10)	116 (CORIN)
ENSP00000277541	NOTCH1	394	0.002	975 (SHH)	385 (JAG2)
ENSP00000379204	BMP7	366	0	974 (SHH)	105 (TGFB2)
ENSP00000295731	IHH	114	0.002	969 (TGFB2)	436 (SHH)
ENSP00000256646	NOTCH2	107	0	966 (JAG2)	376 (JAG2)
ENSP00000264568	BMPR1B	2	0.016	944 (SOX9)	451 (ACVR1C)
ENSP00000366534	FOXH1	120	0.014	913 (FOXF2)	103 (FOXF2)
ENSP00000355192	CACNA1S	107	0.018	907 (CACNA1H)	226 (CACNA1H)

### 3.3 Analysis of the PPIs used to identify candidate genes in graph-based method

As mentioned in Section 2.1, the PPIs used in this study are not all validated by experiments, *i*.*e*., they are not very reliable. However, for wide selection of candidate genes of reproduction, some interactions which are not validated by experiments but can be found evidences in other ways should also be considered, thereby finding additional clues on the identification of novel reproduction-related genes. This section gave the statistical results of the PPIs used in the graph-based method to discover candidate genes of reproduction.

According to the graph-based method, all shortest paths connecting any pair of reproduction-related genes were searched in *G*. Since the graph-based method finally produced 129 genes, we extracted the paths among the aforementioned shortest paths such that each of them contained at least one member of the 129 candidate genes as inner nodes. 877 PPIs were involved in these paths and are provided in S5 Information. It is surprising that 639 (639/877 = 72.86%) interactions have been validated by experiments, which was much higher than the ratio of experimentally verified human PPIs and total human PPIs reported in STRING (86,854/1,640,707 = 5.30%). It is indicated that the candidate genes obtained by the graph-based method are quite reliable. Besides, a same number of PPIs that were not verified by experiments also gave contribution to discover new candidate genes for reproduction, which may provide new clues to study reproduction.

### 3.4 The functional difference between novel and known reproduction genes

To fairly compare the functions of the 21 novel reproduction genes and 115 known reproduction genes and avoid the effect of GO hierarchical structures, we analyzed the count distribution of the 21 novel genes and 115 known genes on level 3, 4 and 5 GO BP (Biological Process), respectively. On each level, the 21 novel genes and 115 known genes were mapped onto the same level GO BP terms and therefore, the GO hierarchical structures will be the same. Then, we used R package goProfiles [51,52] to calculate the significant p value of the functional annotation distributions of the 21 novel genes and 115 known genes that were the same. The p values of GO BP level 3, 4 and 5 terms were 0.0011, 0.0007 and 0.0008, respectively. The results are provided in the S6 Information. This means that the function annotations of the 21 novel genes and 115 known genes were different. The 21 genes include novel information that was not represented by 115 known genes.

We also performed Gene Ontology (GO) term and KEGG pathway analyses of the 21 significant candidate genes using DAVID (Database for Annotation, Visualization and Integrated Discovery) [53]. The enrichment results of 21 novel reproduction genes and 115 known reproduction genes can be found in the S7 Information and S8 Information, respectively.

GO analysis revealed that the 21 novel reproduction genes have significantly enriched functions in cell proliferation, cell differentiation, pattern specification and development. Comparatively, the known reproduction genes were also enriched in differentiation and development functions, but more specific to reproduction (e.g. reproductive developmental process, gamete generation and male gonad development). Furthermore, the novel reproduction genes and the known reproduction genes share several significant GO terms, including cellular process involved in reproduction, developmental process involved in reproduction and single organism reproductive process in the level 3; organ development, anatomical structure morphogenesis and regulation of cell differentiation in the level 4; embryo development, nervous system development and pattern specification process in the level 5. These results suggested the potential roles of novel reproduction genes in the reproduction processes such as gamete generation and embryonic development.

KEGG pathway analysis revealed that the 21 novel reproduction genes were enriched in TGF-β signaling (hsa04350) and cytokine-cytokine receptor interaction pathway (hsa04060). TGF-β (transforming growth factor β) superfamily members, such as bone morphogenetic proteins (BMPs), growth and differentiation factors (GDFs), anti-Müllerian hormone (AMH), Activin, Nodal and TGFβs, were secreted cytokines that involved in a number of important physiological processes in reproduction including the maintenance of stem cell pluripotency [54,55], germ cell development [56,57] and embryonic development [58–60]. Significant amount of activated TGF-β family member proteins were detected in both testis and placenta, and they were reported to regulate male spermatogenesis [56,61] as well as female pregnancy [62,63]. The BMP / Noggin signaling is powerful in controlling ES cell differentiation. BMP2 was reported to control the differentiation of embryonic stem cells into cells with the properties of extra-embryonic endoderm, and Noggin was the antagonist of BMP and blocked this form of differentiation and induced the appearance of a novel cell type that could give rise to neural precursors [64]. In our study, both the reference and the candidate genes show significant enrichment in the TGF-β signaling pathway (9 significant candidate genes shared this pathway: ACVR1, ACVR2A, INHBE, TGFBR1, TGFBR2, BMPR2, BMP4, BMP7, BMPR1B).

Other candidate genes included two Notch proteins (NOTCH1 and NOTCH2), forkhead box protein A1 (FOXA1) and H1 (FOXH1), fibroblast growth factor 4 (FGF4) and receptor 1 (FGFR1), T-box 5 (TBX5), Indian hedgehog (IHH), slit homolog 1 (SLIT1), calcium channel, voltage-dependent, L type, alpha 1S subunit (CACNA1S), nuclear receptor subfamily 0, group B, member 2 (NR0B2) and coagulation factor X (F10). Previous studies revealed part of their roles in reproduction and embryoic development. First, the Notch pathway was shown to play important roles in controlling stem cell proliferation and differentiation [65–67], which is essential in embryonic development. Other studies indicated that Notch pathway were also important for male spermatogenesis [68,69] and female oogenesis [70,71]. Then, both FOXA1 and FOXH1 are important transcription factors. FOXA1 was reported to regulate the differentiation and development of epithelial cells and ducts [72–74], while Foxh1 was shown to control the expression of many genes including Smad and Mixl1 in mouse and xenopus, and functions in patterning the early embryo [75,76]. The FGF signaling pathway is responsible for multiple events during embryo development, such as axial elongation [77] and somitogenesis [78]. Furthermore, they play diverse roles in the male reproductive system. For example, FGFR1 was also shown to be highly expressed in the male testis and maintain the spermatogonia in the undifferentiated state [79]. TBX5, this T-box transcription factor was reported to be closely related to embryonic heart and limb development and mutation of TBX5 could lead to Holt-Oram syndrome [80,81]. IHH, this Indian hedgehog signaling molecule is mainly involved in the chondcrocyte proliferation, differentiation and maturation process [82,83], which is crucial to the bone development and morphogenesis. SLIT1, this gene is highly expressed during embryonic development, mainly in the midline, hypochord, telencephalon and hindbrain with the established roles in axon guidance and cell migration [84,85]. These lines of evidence are consistent with our prediction. Other three genes predicted in our study were CACNA1S, NR0B2 and F10. CACNA1S encodes one subunits of voltage-dependent calcium channel in skeletal muscle cells and its mutation have been associated with malignant hyperthermia [86] and periodic paralysis [87]. NR0B2 is a unique nuclear receptor, which only has ligand-binding domain but no DNA-binding domain. Previous studies showed that NR0B2 interacted with several transcription factors and inhibited their function [88], and it was tightly linked to human diseases such cancer, diabetes and obesity [89]. F10 is the vitamin K-dependent coagulation factor X of the blood coagulation cascade and it also play roles in host defense and innate immunity activation [90]. So far no experimental evidence indicated that these three genes had reproduction-related functions, and further investigations are required to explore their roles in human reproduction.

## Conclusion

This work contributed to the elucidation of the reproductive process by discovering novel human reproduction-related genes. Based on the known reproduction-related genes, PPIs, sequence similarity and interaction confidence score, new candidate genes were identified. Many of these newly identified genes were supported by latest literatures. It is hoped that these findings will guide investigators to confirm novel reproduction-related genes with *in vivo* and *in vitro* experiments.

## Supporting Information

S1 Information The 115 known reproduction-related genes. (DOCX)

S2 Information 406 shortest path genes with betweenness greater than zero and their Permutation FDRs. (DOCX)

S3 Information 129 candidate genes obtained by graph-based method and their maximum alignment scores to reproduction-related gene. (DOCX)

S4 Information 27 candidate genes filtered by the second step of hybrid method and their maximum interaction score to reproduction-related gene. (DOCX)

S5 Information The protein-protein interactions used to identify 129 candidate genes for reproduction. (XLSX)

S6 Information The functional annotation distributions of the 21 novel reproduction genes and 115 known reproduction genes on GO BP level 3, 4 and 5. (XLSX)

S7 Information GO and KEGG enrichment results of 21 novel reproduction genes. (XLSX)

S8 Information GO and KEGG enrichment results of 115 known reproduction genes. (XLSX)
