# Supplementary material for: A Hybrid Computational Method for the Discovery of Novel Reproduction-Related Genes
Source: PLoS One. 2015 Mar 13;10(3):e0117090. doi: 10.1371/journal.pone.0117090 (PMC4358884; doi:10.1371/journal.pone.0117090)
Supplement: S1 Information — (DOCX) [file pone.0117090.s001.docx]

**Online Supporting Information S1.** 115 known reproduction-related genes

GDF9

ZP3

RBMY1B

KITLG

UTF1

PROK2

EIF2B4

SPIRE2

SLC22A16

NLRP14

SLIT2

HNF1B

SRY

TGFB2

ZP4

TIMP1

CBX2

SOX3

WNT5A

CRH

ZFP42

RPL10L

ACVR1B

DNAJC19

STRA13

FSHR

WNT4

UBAP2L

SPIRE1

RPL39L

CACNA1H

ZP2

GALNTL5

TNP1

SGOL1

SLIT3

SOX9

CRHBP

GATA1

AMH

CRIP1

PKD1

NIPBL

ENDOU

RACGAP1

DPY19L2

XRN2

PTPN11

ACVR1C

WNT2B

TDRP

VDR

GLRA1

RHOXF1

CYP27B1

TBX3

HPGD

NODAL

MOV10L1

STRA6

NR5A1

NPM2

MYCBP

GATA4

EIF2B5

BOLL

MAMLD1

SPATA25

GHSR

INHBA

KIT

TFAP2C

TGFB3

SMC3

CALCA

FOXF2

FGF8

KLK14

MYCBPAP

SUN5

GATA6

BPY2

ACR

SYT6

GHRL

SHH

CD9

ERBB4

WT1

SFRP1

FGF9

APITD1

STOX2

CHD7

SRD5A2

FOXL2

NANOS3

TAF1L

SYCP3

IZUMO1

EIF2B2

FANCM

HILS1

C1QBP

NR0B1

TCFL5

CORIN

SERPINA10

ROBO2

JAG2

IRX5

SALL1

AKR1C3

PRDM9

MMP23A
