# Supplementary material for: A Hybrid Computational Method for the Discovery of Novel Reproduction-Related Genes
Source: PLoS One. 2015 Mar 13;10(3):e0117090. doi: 10.1371/journal.pone.0117090 (PMC4358884; doi:10.1371/journal.pone.0117090)
Supplement: S2 Information — (DOCX) [file pone.0117090.s002.docx]

**Online Supporting Information S2.** 406 shortest path genes with betweenness greater than zero and their permutation FDRs

| **Ensembl ID** | **Gene symbol** | **Betweenness** | **Permutation FDR** |
| --- | --- | --- | --- |
| ENSP00000229307 | NANOG | 107 | 0 |
| ENSP00000241416 | ACVR2A | 266 | 0 |
| ENSP00000245255 | PIWIL1 | 107 | 0 |
| ENSP00000247182 | SIX1 | 107 | 0 |
| ENSP00000250003 | MYOD1 | 420 | 0 |
| ENSP00000252971 | MNX1 | 107 | 0 |
| ENSP00000253122 | SLC6A8 | 107 | 0 |
| ENSP00000256646 | NOTCH2 | 107 | 0 |
| ENSP00000262965 | TCF3 | 320 | 0 |
| ENSP00000263754 | KAT2B | 166 | 0 |
| ENSP00000266058 | SLIT1 | 212 | 0 |
| ENSP00000266646 | INHBE | 199 | 0 |
| ENSP00000270538 | TIMM44 | 107 | 0 |
| ENSP00000278616 | ATM | 206 | 0 |
| ENSP00000295987 | SYN1 | 107 | 0 |
| ENSP00000296145 | TDGF1 | 107 | 0 |
| ENSP00000299293 | FRS2 | 261 | 0 |
| ENSP00000300177 | GREM1 | 20 | 0 |
| ENSP00000303325 | TACR3 | 1 | 0 |
| ENSP00000305692 | GAA | 107 | 0 |
| ENSP00000305769 | SMAD1 | 448 | 0 |
| ENSP00000323300 | SPAG5 | 107 | 0 |
| ENSP00000323421 | SMC1A | 206 | 0 |
| ENSP00000323659 | KDM3A | 107 | 0 |
| ENSP00000332194 | HIST2H2AC | 107 | 0 |
| ENSP00000333097 | FIGLA | 311 | 0 |
| ENSP00000333950 | FMN1 | 213 | 0 |
| ENSP00000347198 | SRGAP1 | 312 | 0 |
| ENSP00000352721 | DNM2 | 107 | 0 |
| ENSP00000359423 | MTM1 | 107 | 0 |
| ENSP00000363115 | FGR | 107 | 0 |
| ENSP00000363826 | FZD8 | 107 | 0 |
| ENSP00000379204 | BMP7 | 366 | 0 |
| ENSP00000380280 | FGFR1 | 437 | 0 |
| ENSP00000396439 | RING1 | 107 | 0 |
| ENSP00000419494 | RYBP | 107 | 0 |
| ENSP00000222256 | RAB3A | 107 | 0.002 |
| ENSP00000230658 | ISL1 | 1 | 0.002 |
| ENSP00000254122 | FSHB | 111 | 0.002 |
| ENSP00000260653 | SIX3 | 8 | 0.002 |
| ENSP00000263253 | EP300 | 1195 | 0.002 |
| ENSP00000263640 | ACVR1 | 136 | 0.002 |
| ENSP00000264839 | RIMS1 | 107 | 0.002 |
| ENSP00000277541 | NOTCH1 | 394 | 0.002 |
| ENSP00000290953 | AGRP | 4 | 0.002 |
| ENSP00000295731 | IHH | 114 | 0.002 |
| ENSP00000326699 | CLGN | 3 | 0.002 |
| ENSP00000341551 | SMAD4 | 512 | 0.002 |
| ENSP00000343745 | DICER1 | 107 | 0.002 |
| ENSP00000346012 | RPL36AL | 105 | 0.002 |
| ENSP00000349465 | PICK1 | 107 | 0.002 |
| ENSP00000356623 | CITED2 | 107 | 0.002 |
| ENSP00000365663 | NPPA | 107 | 0.002 |
| ENSP00000379213 | PTHLH | 17 | 0.002 |
| ENSP00000403536 | GAMT | 107 | 0.002 |
| ENSP00000168712 | FGF4 | 107 | 0.004 |
| ENSP00000253401 | ARHGEF9 | 107 | 0.004 |
| ENSP00000268171 | FURIN | 107 | 0.004 |
| ENSP00000303019 | GPHN | 107 | 0.004 |
| ENSP00000304669 | CTNNA1 | 190 | 0.004 |
| ENSP00000317333 | NEUROG2 | 1 | 0.004 |
| ENSP00000345206 | RBPJ | 107 | 0.004 |
| ENSP00000363708 | BMPR2 | 127 | 0.004 |
| ENSP00000367756 | UNC13B | 107 | 0.004 |
| ENSP00000373952 | FANCA | 212 | 0.004 |
| ENSP00000256383 | EIF2S1 | 315 | 0.006 |
| ENSP00000262160 | SMAD2 | 600 | 0.006 |
| ENSP00000267859 | BNIP2 | 157 | 0.006 |
| ENSP00000309831 | SNUPN | 205 | 0.006 |
| ENSP00000319060 | CAMK2G | 107 | 0.006 |
| ENSP00000364133 | TGFBR1 | 217 | 0.006 |
| ENSP00000368401 | PAX6 | 107 | 0.006 |
| ENSP00000327758 | NKX2-5 | 206 | 0.008 |
| ENSP00000328181 | NOG | 17 | 0.008 |
| ENSP00000364709 | F10 | 107 | 0.008 |
| ENSP00000261349 | LRP6 | 258 | 0.01 |
| ENSP00000264039 | GPC1 | 17 | 0.01 |
| ENSP00000266987 | TARBP2 | 3 | 0.01 |
| ENSP00000303706 | CDC25A | 107 | 0.01 |
| ENSP00000336790 | ATF4 | 290 | 0.01 |
| ENSP00000359290 | DR1 | 2 | 0.01 |
| ENSP00000386896 | ITGA6 | 211 | 0.01 |
| ENSP00000232424 | HES1 | 81 | 0.012 |
| ENSP00000245451 | BMP4 | 172 | 0.012 |
| ENSP00000265708 | ADAM2 | 3 | 0.012 |
| ENSP00000359531 | GTF2B | 107 | 0.012 |
| ENSP00000231061 | SPARC | 102 | 0.014 |
| ENSP00000260363 | KIF23 | 3 | 0.014 |
| ENSP00000264426 | GRIA2 | 4 | 0.014 |
| ENSP00000338207 | LMO1 | 5 | 0.014 |
| ENSP00000366534 | FOXH1 | 120 | 0.014 |
| ENSP00000264110 | ATF2 | 107 | 0.016 |
| ENSP00000264568 | BMPR1B | 2 | 0.016 |
| ENSP00000332353 | PTCH1 | 109 | 0.016 |
| ENSP00000337736 | AKAP1 | 6 | 0.016 |
| ENSP00000344352 | ATF3 | 312 | 0.016 |
| ENSP00000349320 | CACNA2D1 | 107 | 0.016 |
| ENSP00000217086 | SALL4 | 2 | 0.018 |
| ENSP00000254227 | NR0B2 | 232 | 0.018 |
| ENSP00000355192 | CACNA1S | 107 | 0.018 |
| ENSP00000364976 | CKS2 | 1 | 0.02 |
| ENSP00000249598 | GDF2 | 4 | 0.022 |
| ENSP00000262999 | UCP1 | 64 | 0.022 |
| ENSP00000358716 | DDX20 | 208 | 0.024 |
| ENSP00000237527 | GHRH | 7 | 0.026 |
| ENSP00000260433 | CYP19A1 | 1 | 0.026 |
| ENSP00000302961 | HSPA4 | 107 | 0.026 |
| ENSP00000333203 | SERPINA5 | 105 | 0.026 |
| ENSP00000349959 | RICTOR | 104 | 0.026 |
| ENSP00000298552 | TSC1 | 106 | 0.028 |
| ENSP00000351905 | TGFBR2 | 1 | 0.028 |
| ENSP00000360687 | PTGDS | 106 | 0.028 |
| ENSP00000250448 | FOXA1 | 5 | 0.034 |
| ENSP00000309913 | TBX5 | 2 | 0.034 |
| ENSP00000332973 | SMAD3 | 178 | 0.034 |
| ENSP00000370119 | SMN2 | 105 | 0.034 |
| ENSP00000297338 | RAD21 | 6 | 0.036 |
| ENSP00000299766 | MC4R | 4 | 0.036 |
| ENSP00000302630 | ONECUT1 | 13 | 0.036 |
| ENSP00000262238 | YY1 | 107 | 0.038 |
| ENSP00000234071 | PROC | 105 | 0.04 |
| ENSP00000256759 | FST | 6 | 0.04 |
| ENSP00000344456 | CTNNB1 | 1134 | 0.042 |
| ENSP00000368169 | DVL1 | 159 | 0.042 |
| ENSP00000314458 | CDC42 | 492 | 0.044 |
| ENSP00000325313 | MAP1S | 106 | 0.044 |
| ENSP00000361818 | SDC4 | 2 | 0.044 |
| ENSP00000241651 | MYOG | 106 | 0.046 |
| ENSP00000233156 | TFPI | 2 | 0.048 |
| ENSP00000268053 | CYP11A1 | 80 | 0.05 |
| ENSP00000356771 | F5 | 104 | 0.05 |
| ENSP00000399968 | NCOA2 | 181 | 0.05 |
| ENSP00000271555 | MEF2D | 14 | 0.052 |
| ENSP00000284523 | WNT3A | 2 | 0.052 |
| ENSP00000352561 | CALCR | 121 | 0.052 |
| ENSP00000244007 | PLCG1 | 170 | 0.054 |
| ENSP00000344822 | S100A13 | 8 | 0.054 |
| ENSP00000346440 | TCF4 | 29 | 0.056 |
| ENSP00000358335 | MAP3K7 | 5 | 0.056 |
| ENSP00000176195 | SCT | 121 | 0.058 |
| ENSP00000264690 | KLKB1 | 8 | 0.058 |
| ENSP00000272190 | REN | 2 | 0.058 |
| ENSP00000298171 | TSHR | 8 | 0.058 |
| ENSP00000370421 | PDX1 | 23 | 0.062 |
| ENSP00000287934 | FZD1 | 104 | 0.064 |
| ENSP00000312987 | HNF4A | 71 | 0.064 |
| ENSP00000354621 | SMURF1 | 91 | 0.066 |
| ENSP00000223023 | WASL | 139 | 0.07 |
| ENSP00000262033 | PTGES3 | 2 | 0.07 |
| ENSP00000356438 | PTGS2 | 212 | 0.07 |
| ENSP00000381098 | GRIP1 | 4 | 0.07 |
| ENSP00000293549 | WNT1 | 47 | 0.072 |
| ENSP00000346879 | NKX2-1 | 105 | 0.072 |
| ENSP00000260270 | FDX1 | 40 | 0.074 |
| ENSP00000261205 | SYT1 | 8 | 0.074 |
| ENSP00000358151 | HIST2H2BE | 2 | 0.074 |
| ENSP00000376765 | PIAS3 | 4 | 0.074 |
| ENSP00000283147 | BMP6 | 8 | 0.076 |
| ENSP00000287038 | RPL30 | 105 | 0.076 |
| ENSP00000353393 | F8 | 108 | 0.076 |
| ENSP00000276414 | GNRH1 | 105 | 0.078 |
| ENSP00000284384 | PRKCA | 103 | 0.08 |
| ENSP00000364094 | ITGB1 | 211 | 0.08 |
| ENSP00000262584 | RPL8 | 106 | 0.082 |
| ENSP00000262735 | PPARA | 43 | 0.082 |
| ENSP00000301843 | CTTN | 106 | 0.082 |
| ENSP00000405574 | TBL1XR1 | 2 | 0.082 |
| ENSP00000410294 | FGFR2 | 12 | 0.082 |
| ENSP00000206249 | ESR1 | 469 | 0.084 |
| ENSP00000262158 | SMAD7 | 68 | 0.084 |
| ENSP00000366482 | FXN | 26 | 0.086 |
| ENSP00000233057 | EIF2AK2 | 3 | 0.09 |
| ENSP00000262187 | RHEB | 4 | 0.09 |
| ENSP00000293195 | FDXR | 40 | 0.09 |
| ENSP00000312652 | LEP | 374 | 0.092 |
| ENSP00000321106 | TAC1 | 1 | 0.092 |
| ENSP00000375921 | PAX3 | 1 | 0.092 |
| ENSP00000396219 | MEF2C | 106 | 0.092 |
| ENSP00000349547 | RASSF1 | 106 | 0.094 |
| ENSP00000352608 | RYR1 | 107 | 0.096 |
| ENSP00000219476 | TSC2 | 72 | 0.098 |
| ENSP00000265171 | EGF | 204 | 0.098 |
| ENSP00000363804 | KLF4 | 94 | 0.098 |
| ENSP00000324806 | GSK3B | 383 | 0.102 |
| ENSP00000329357 | SP1 | 280 | 0.104 |
| ENSP00000265023 | KNG1 | 109 | 0.108 |
| ENSP00000371138 | FKBP1A | 148 | 0.112 |
| ENSP00000256010 | NTS | 1 | 0.114 |
| ENSP00000323588 | SOX2 | 11 | 0.116 |
| ENSP00000297494 | NOS3 | 107 | 0.122 |
| ENSP00000261769 | CDH1 | 107 | 0.124 |
| ENSP00000309597 | MAP3K11 | 7 | 0.124 |
| ENSP00000340330 | KAT5 | 105 | 0.124 |
| ENSP00000264708 | POMC | 211 | 0.126 |
| ENSP00000302150 | PRL | 181 | 0.126 |
| ENSP00000377969 | GTF2F1 | 1 | 0.126 |
| ENSP00000264606 | HDAC4 | 3 | 0.128 |
| ENSP00000264867 | PPARGC1A | 112 | 0.128 |
| ENSP00000378338 | GIT1 | 21 | 0.13 |
| ENSP00000371432 | PRLR | 175 | 0.132 |
| ENSP00000373715 | DCP2 | 7 | 0.132 |
| ENSP00000241014 | MAPK8IP1 | 7 | 0.136 |
| ENSP00000302234 | CCL11 | 1 | 0.138 |
| ENSP00000366607 | SF1 | 1 | 0.138 |
| ENSP00000395498 | PAX8 | 98 | 0.138 |
| ENSP00000220592 | AGO2 | 104 | 0.14 |
| ENSP00000225916 | KAT2A | 2 | 0.14 |
| ENSP00000249071 | RAC2 | 4 | 0.14 |
| ENSP00000352516 | DNMT1 | 107 | 0.14 |
| ENSP00000308541 | F2 | 199 | 0.146 |
| ENSP00000374354 | EXOSC8 | 99 | 0.146 |
| ENSP00000419692 | RXRA | 85 | 0.146 |
| ENSP00000278568 | PAK1 | 55 | 0.148 |
| ENSP00000294339 | TAL1 | 69 | 0.152 |
| ENSP00000325690 | CARM1 | 37 | 0.152 |
| ENSP00000254976 | SNAP25 | 107 | 0.16 |
| ENSP00000295600 | MITF | 96 | 0.16 |
| ENSP00000330393 | LEPR | 25 | 0.166 |
| ENSP00000361275 | PLK3 | 70 | 0.166 |
| ENSP00000329380 | GP1BA | 195 | 0.168 |
| ENSP00000360141 | GNAS | 6 | 0.168 |
| ENSP00000258743 | IL6 | 106 | 0.17 |
| ENSP00000296029 | PF4 | 1 | 0.174 |
| ENSP00000307863 | U2AF2 | 1 | 0.18 |
| ENSP00000358595 | CGA | 6 | 0.182 |
| ENSP00000380227 | ITGA4 | 231 | 0.184 |
| ENSP00000287820 | PPARG | 105 | 0.192 |
| ENSP00000367207 | MYC | 230 | 0.194 |
| ENSP00000308938 | PLG | 106 | 0.196 |
| ENSP00000313950 | AURKB | 31 | 0.196 |
| ENSP00000347733 | TRRAP | 4 | 0.196 |
| ENSP00000237837 | FGF23 | 73 | 0.198 |
| ENSP00000313829 | KHDRBS1 | 5 | 0.206 |
| ENSP00000257408 | KLB | 73 | 0.21 |
| ENSP00000368683 | EDN1 | 2 | 0.21 |
| ENSP00000263025 | MAPK3 | 6 | 0.212 |
| ENSP00000282091 | PTH | 38 | 0.216 |
| ENSP00000362795 | CXCR3 | 1 | 0.222 |
| ENSP00000330341 | SOCS3 | 6 | 0.224 |
| ENSP00000350283 | BRCA1 | 242 | 0.226 |
| ENSP00000242152 | NPY | 2 | 0.228 |
| ENSP00000252622 | LSM7 | 99 | 0.228 |
| ENSP00000396127 | RAN | 199 | 0.228 |
| ENSP00000309503 | YWHAZ | 195 | 0.23 |
| ENSP00000222139 | EPOR | 5 | 0.238 |
| ENSP00000368880 | FOXO1 | 8 | 0.24 |
| ENSP00000314151 | KLK3 | 2 | 0.244 |
| ENSP00000338799 | IL6ST | 21 | 0.244 |
| ENSP00000348577 | RANGAP1 | 152 | 0.246 |
| ENSP00000384675 | SOS1 | 81 | 0.248 |
| ENSP00000323929 | A2M | 8 | 0.25 |
| ENSP00000338548 | FGF1 | 72 | 0.25 |
| ENSP00000269571 | ERBB2 | 272 | 0.254 |
| ENSP00000292408 | FGFR4 | 75 | 0.254 |
| ENSP00000262320 | AXIN1 | 93 | 0.258 |
| ENSP00000228307 | PXN | 231 | 0.262 |
| ENSP00000321999 | PTH1R | 1 | 0.262 |
| ENSP00000243050 | NR4A1 | 134 | 0.264 |
| ENSP00000354586 | GLI2 | 7 | 0.264 |
| ENSP00000281950 | GEMIN6 | 2 | 0.266 |
| ENSP00000221930 | TGFB1 | 94 | 0.27 |
| ENSP00000256592 | TSHB | 6 | 0.274 |
| ENSP00000265165 | LEF1 | 91 | 0.274 |
| ENSP00000315615 | AKAP5 | 6 | 0.276 |
| ENSP00000324897 | UBE2I | 177 | 0.28 |
| ENSP00000265563 | PRKAR2A | 6 | 0.284 |
| ENSP00000228837 | FGF6 | 3 | 0.288 |
| ENSP00000301633 | BIRC5 | 35 | 0.288 |
| ENSP00000371067 | JAK2 | 223 | 0.294 |
| ENSP00000309845 | HRAS | 130 | 0.302 |
| ENSP00000215829 | SNRPD3 | 99 | 0.304 |
| ENSP00000341189 | PTK2 | 170 | 0.304 |
| ENSP00000349467 | CALM1 | 107 | 0.304 |
| ENSP00000339007 | GRB2 | 719 | 0.306 |
| ENSP00000235090 | WDR77 | 1 | 0.318 |
| ENSP00000360266 | JUN | 167 | 0.318 |
| ENSP00000230354 | TBP | 212 | 0.32 |
| ENSP00000299543 | CTDP1 | 1 | 0.32 |
| ENSP00000212015 | SIRT1 | 112 | 0.322 |
| ENSP00000229794 | MAPK14 | 3 | 0.324 |
| ENSP00000365016 | IRS2 | 5 | 0.324 |
| ENSP00000216911 | AURKA | 10 | 0.33 |
| ENSP00000334122 | FGF3 | 2 | 0.33 |
| ENSP00000346839 | FN1 | 157 | 0.33 |
| ENSP00000267163 | RB1 | 132 | 0.332 |
| ENSP00000268182 | IQGAP1 | 120 | 0.336 |
| ENSP00000225577 | RPS6KB1 | 4 | 0.338 |
| ENSP00000223095 | SERPINE1 | 11 | 0.344 |
| ENSP00000368104 | BMP2 | 14 | 0.344 |
| ENSP00000318297 | RUVBL1 | 14 | 0.346 |
| ENSP00000319169 | PRMT5 | 1 | 0.38 |
| ENSP00000348708 | UPF2 | 8 | 0.38 |
| ENSP00000361850 | PLAU | 10 | 0.39 |
| ENSP00000263967 | PIK3CA | 29 | 0.402 |
| ENSP00000339992 | MYB | 42 | 0.408 |
| ENSP00000309103 | BAD | 93 | 0.412 |
| ENSP00000249636 | PIAS1 | 14 | 0.414 |
| ENSP00000350941 | SRC | 365 | 0.422 |
| ENSP00000264657 | STAT3 | 563 | 0.424 |
| ENSP00000329623 | BCL2 | 314 | 0.428 |
| ENSP00000356505 | NCF2 | 28 | 0.436 |
| ENSP00000369757 | RPS6 | 10 | 0.436 |
| ENSP00000280892 | EIF4E | 22 | 0.442 |
| ENSP00000222254 | PIK3R2 | 2 | 0.452 |
| ENSP00000283195 | RANBP2 | 47 | 0.458 |
| ENSP00000266970 | CDK2 | 64 | 0.466 |
| ENSP00000345571 | E2F1 | 41 | 0.478 |
| ENSP00000302967 | HDAC3 | 2 | 0.484 |
| ENSP00000364893 | ARHGEF7 | 34 | 0.484 |
| ENSP00000354394 | STAT1 | 100 | 0.488 |
| ENSP00000317714 | STX4 | 1 | 0.492 |
| ENSP00000302486 | MAP2K1 | 15 | 0.496 |
| ENSP00000306245 | FOS | 20 | 0.496 |
| ENSP00000363676 | RPL11 | 184 | 0.5 |
| ENSP00000387662 | GCG | 122 | 0.502 |
| ENSP00000353483 | MAPK8 | 185 | 0.504 |
| ENSP00000339527 | FOXO3 | 9 | 0.514 |
| ENSP00000274026 | CCNA2 | 65 | 0.518 |
| ENSP00000320935 | SLC2A4 | 1 | 0.52 |
| ENSP00000340691 | EIF4EBP1 | 2 | 0.52 |
| ENSP00000226218 | VTN\|SEBOX | 1 | 0.528 |
| ENSP00000227507 | CCND1 | 153 | 0.528 |
| ENSP00000376076 | SUMO1 | 22 | 0.548 |
| ENSP00000363822 | AR | 62 | 0.556 |
| ENSP00000251849 | RAF1 | 64 | 0.568 |
| ENSP00000268712 | NCOR1 | 2 | 0.568 |
| ENSP00000282561 | GJA1 | 8 | 0.57 |
| ENSP00000354558 | MTOR | 103 | 0.57 |
| ENSP00000268035 | IGF1R | 34 | 0.572 |
| ENSP00000307046 | SDC2 | 2 | 0.572 |
| ENSP00000342793 | PLD1 | 73 | 0.58 |
| ENSP00000359345 | RPL5 | 17 | 0.582 |
| ENSP00000269305 | TP53 | 1802 | 0.594 |
| ENSP00000372023 | CHEK2 | 68 | 0.6 |
| ENSP00000292303 | CCR5 | 1 | 0.608 |
| ENSP00000300574 | CRK | 23 | 0.608 |
| ENSP00000300093 | PLK1 | 111 | 0.632 |
| ENSP00000306512 | IL8 | 2 | 0.636 |
| ENSP00000391592 | PTPN6 | 2 | 0.638 |
| ENSP00000358997 | IRAK1 | 9 | 0.646 |
| ENSP00000361021 | PTEN | 83 | 0.648 |
| ENSP00000255465 | CCNA1 | 23 | 0.658 |
| ENSP00000310491 | ARHGAP1 | 1 | 0.668 |
| ENSP00000321656 | CDC25C | 129 | 0.672 |
| ENSP00000299421 | ILK | 15 | 0.674 |
| ENSP00000304895 | IRS1 | 116 | 0.676 |
| ENSP00000400175 | RHOA | 5 | 0.692 |
| ENSP00000222812 | STX1A | 98 | 0.694 |
| ENSP00000333001 | RBM8A | 1 | 0.696 |
| ENSP00000263341 | IL1B | 15 | 0.702 |
| ENSP00000368438 | PCNA | 27 | 0.708 |
| ENSP00000227378 | HSPA8 | 4 | 0.712 |
| ENSP00000258682 | CAMK2B | 52 | 0.712 |
| ENSP00000262367 | CREBBP | 42 | 0.712 |
| ENSP00000274376 | RASA1 | 3 | 0.716 |
| ENSP00000348986 | INS-IGF2 | 83 | 0.716 |
| ENSP00000335153 | HSP90AA1 | 40 | 0.726 |
| ENSP00000226574 | NFKB1 | 20 | 0.732 |
| ENSP00000352400 | NUP214 | 1 | 0.738 |
| ENSP00000276201 | UPF3B | 1 | 0.748 |
| ENSP00000288986 | NCK1 | 6 | 0.752 |
| ENSP00000269321 | ARHGDIA | 22 | 0.754 |
| ENSP00000361066 | NCOA3 | 5 | 0.762 |
| ENSP00000278916 | CHEK1 | 39 | 0.776 |
| ENSP00000270202 | AKT1 | 434 | 0.778 |
| ENSP00000003084 | CFTR | 98 | 0.786 |
| ENSP00000294172 | NXF1 | 1 | 0.786 |
| ENSP00000362649 | HDAC1 | 107 | 0.788 |
| ENSP00000361125 | VEGFA | 3 | 0.79 |
| ENSP00000263923 | KDR | 2 | 0.792 |
| ENSP00000343204 | JAK1 | 3 | 0.802 |
| ENSP00000417281 | MDM2 | 255 | 0.806 |
| ENSP00000312995 | CLSPN | 5 | 0.808 |
| ENSP00000346300 | CRKL | 3 | 0.818 |
| ENSP00000348461 | RAC1 | 222 | 0.83 |
| ENSP00000228872 | CDKN1B | 51 | 0.846 |
| ENSP00000264033 | CBL | 115 | 0.846 |
| ENSP00000401303 | SHC1 | 38 | 0.848 |
| ENSP00000262613 | SLC9A3R1 | 94 | 0.85 |
| ENSP00000280357 | IL18 | 2 | 0.856 |
| ENSP00000284981 | APP | 22 | 0.86 |
| ENSP00000387699 | CREB1 | 60 | 0.864 |
| ENSP00000215832 | MAPK1 | 41 | 0.868 |
| ENSP00000338018 | HIF1A | 81 | 0.888 |
| ENSP00000244741 | CDKN1A | 21 | 0.89 |
| ENSP00000360683 | PTPN1 | 8 | 0.892 |
| ENSP00000326366 | PSEN1 | 21 | 0.896 |
| ENSP00000361626 | YBX1 | 47 | 0.902 |
| ENSP00000274335 | PIK3R1 | 4 | 0.906 |
| ENSP00000302269 | VAV1 | 47 | 0.912 |
| ENSP00000275493 | EGFR | 436 | 0.914 |
| ENSP00000303830 | INSR | 7 | 0.924 |
| ENSP00000261799 | PDGFRB | 55 | 0.932 |
| ENSP00000229135 | IFNG | 2 | 0.944 |
| ENSP00000221494 | SF3A2 | 96 | 0.952 |
| ENSP00000365891 | WAS | 1 | 0.952 |
| ENSP00000011653 | CD4 | 3 | 0.958 |
| ENSP00000304903 | CD2BP2 | 46 | 0.966 |
| ENSP00000358490 | CD2 | 46 | 0.97 |
| ENSP00000384273 | RELA | 29 | 0.984 |
| ENSP00000359206 | BTRC | 7 | 0.99 |
| ENSP00000046794 | LCP2 | 35 | 0.992 |
| ENSP00000337825 | LCK | 46 | 0.994 |
| ENSP00000226730 | IL2 | 8 | 0.998 |
| ENSP00000344818 | UBC | 242 | 1 |
| ENSP00000357656 | FYN | 1 | 1 |
