# Supplementary material for: A Hybrid Computational Method for the Discovery of Novel Reproduction-Related Genes
Source: PLoS One. 2015 Mar 13;10(3):e0117090. doi: 10.1371/journal.pone.0117090 (PMC4358884; doi:10.1371/journal.pone.0117090)
Supplement: S3 Information — (DOCX) [file pone.0117090.s003.docx]

**Online Supporting Information S3.** 129 candidate genes obtained by graph-based method and their maximum alignment scores to reproduction-related gene

| **Ensembl ID** | **Gene symbol** | **Maximum alignment score to reproduction-related gene** |
| --- | --- | --- |
| ENSP00000266058 | SLIT1 | 1995 |
| ENSP00000364133 | TGFBR1 | 682 |
| ENSP00000264568 | BMPR1B | 451 |
| ENSP00000263640 | ACVR1 | 439 |
| ENSP00000295731 | IHH | 436 |
| ENSP00000277541 | NOTCH1 | 385 |
| ENSP00000256646 | NOTCH2 | 376 |
| ENSP00000217086 | SALL4 | 314 |
| ENSP00000309913 | TBX5 | 244 |
| ENSP00000380280 | FGFR1 | 239 |
| ENSP00000262238 | YY1 | 238 |
| ENSP00000241416 | ACVR2A | 233 |
| ENSP00000351905 | TGFBR2 | 231 |
| ENSP00000355192 | CACNA1S | 226 |
| ENSP00000363708 | BMPR2 | 194 |
| ENSP00000333203 | SERPINA5 | 181 |
| ENSP00000363115 | FGR | 174 |
| ENSP00000234071 | PROC | 152 |
| ENSP00000266646 | INHBE | 149 |
| ENSP00000254227 | NR0B2 | 129 |
| ENSP00000250448 | FOXA1 | 120 |
| ENSP00000364709 | F10 | 116 |
| ENSP00000249598 | GDF2 | 115 |
| ENSP00000245451 | BMP4 | 105 |
| ENSP00000379204 | BMP7 | 105 |
| ENSP00000168712 | FGF4 | 104 |
| ENSP00000366534 | FOXH1 | 103 |
| ENSP00000363826 | FZD8 | 86 |
| ENSP00000261349 | LRP6 | 85 |
| ENSP00000303325 | TACR3 | 73 |
| ENSP00000323421 | SMC1A | 72 |
| ENSP00000260433 | CYP19A1 | 69 |
| ENSP00000264839 | RIMS1 | 65 |
| ENSP00000347198 | SRGAP1 | 65 |
| ENSP00000263754 | KAT2B | 64 |
| ENSP00000343745 | DICER1 | 64 |
| ENSP00000368401 | PAX6 | 60 |
| ENSP00000319060 | CAMK2G | 56 |
| ENSP00000263253 | EP300 | 54 |
| ENSP00000327758 | NKX2-5 | 53 |
| ENSP00000252971 | MNX1 | 52 |
| ENSP00000367756 | UNC13B | 51 |
| ENSP00000299766 | MC4R | 44 |
| ENSP00000230658 | ISL1 | 42 |
| ENSP00000247182 | SIX1 | 40 |
| ENSP00000229307 | NANOG | 39 |
| ENSP00000264110 | ATF2 | 39 |
| ENSP00000260653 | SIX3 | 36 |
| ENSP00000344456 | CTNNB1 | 35 |
| ENSP00000262999 | UCP1 | 32 |
| ENSP00000268171 | FURIN | 31 |
| ENSP00000302630 | ONECUT1 | 30 |
| ENSP00000305692 | GAA | 30 |
| ENSP00000337736 | AKAP1 | 30 |
| ENSP00000338207 | LMO1 | 30 |
| ENSP00000345206 | RBPJ | 30 |
| ENSP00000231061 | SPARC | 28 |
| ENSP00000296145 | TDGF1 | 28 |
| ENSP00000323300 | SPAG5 | 28 |
| ENSP00000332353 | PTCH1 | 28 |
| ENSP00000256759 | FST | 27 |
| ENSP00000265708 | ADAM2 | 27 |
| ENSP00000270538 | TIMM44 | 27 |
| ENSP00000358716 | DDX20 | 27 |
| ENSP00000403536 | GAMT | 27 |
| ENSP00000260363 | KIF23 | 26 |
| ENSP00000264039 | GPC1 | 26 |
| ENSP00000302961 | HSPA4 | 26 |
| ENSP00000303019 | GPHN | 26 |
| ENSP00000245255 | PIWIL1 | 25 |
| ENSP00000253401 | ARHGEF9 | 25 |
| ENSP00000254122 | FSHB | 25 |
| ENSP00000278616 | ATM | 25 |
| ENSP00000298552 | TSC1 | 25 |
| ENSP00000304669 | CTNNA1 | 25 |
| ENSP00000326699 | CLGN | 25 |
| ENSP00000333950 | FMN1 | 25 |
| ENSP00000349959 | RICTOR | 25 |
| ENSP00000356623 | CITED2 | 25 |
| ENSP00000368169 | DVL1 | 25 |
| ENSP00000373952 | FANCA | 25 |
| ENSP00000256383 | EIF2S1 | 24 |
| ENSP00000264426 | GRIA2 | 24 |
| ENSP00000299293 | FRS2 | 24 |
| ENSP00000303706 | CDC25A | 24 |
| ENSP00000317333 | NEUROG2 | 24 |
| ENSP00000359531 | GTF2B | 24 |
| ENSP00000364976 | CKS2 | 24 |
| ENSP00000365663 | NPPA | 24 |
| ENSP00000396439 | RING1 | 24 |
| ENSP00000222256 | RAB3A | 23 |
| ENSP00000232424 | HES1 | 23 |
| ENSP00000241651 | MYOG | 23 |
| ENSP00000262965 | TCF3 | 23 |
| ENSP00000266987 | TARBP2 | 23 |
| ENSP00000297338 | RAD21 | 23 |
| ENSP00000300177 | GREM1 | 23 |
| ENSP00000309831 | SNUPN | 23 |
| ENSP00000323659 | KDM3A | 23 |
| ENSP00000333097 | FIGLA | 23 |
| ENSP00000359290 | DR1 | 23 |
| ENSP00000360687 | PTGDS | 23 |
| ENSP00000386896 | ITGA6 | 23 |
| ENSP00000237527 | GHRH | 22 |
| ENSP00000250003 | MYOD1 | 22 |
| ENSP00000332194 | HIST2H2AC | 22 |
| ENSP00000332973 | SMAD3 | 22 |
| ENSP00000341551 | SMAD4 | 22 |
| ENSP00000344352 | ATF3 | 22 |
| ENSP00000352721 | DNM2 | 22 |
| ENSP00000290953 | AGRP | 21 |
| ENSP00000336790 | ATF4 | 21 |
| ENSP00000346012 | RPL36AL | 21 |
| ENSP00000361818 | SDC4 | 20 |
| ENSP00000379213 | PTHLH | 20 |
| ENSP00000233156 | TFPI | 0 |
| ENSP00000253122 | SLC6A8 | 0 |
| ENSP00000262160 | SMAD2 | 0 |
| ENSP00000267859 | BNIP2 | 0 |
| ENSP00000295987 | SYN1 | 0 |
| ENSP00000305769 | SMAD1 | 0 |
| ENSP00000314458 | CDC42 | 0 |
| ENSP00000325313 | MAP1S | 0 |
| ENSP00000328181 | NOG | 0 |
| ENSP00000349320 | CACNA2D1 | 0 |
| ENSP00000349465 | PICK1 | 0 |
| ENSP00000359423 | MTM1 | 0 |
| ENSP00000370119 | SMN2 | 0 |
| ENSP00000419494 | RYBP | 0 |
