# Supplementary material for: A Hybrid Computational Method for the Discovery of Novel Reproduction-Related Genes
Source: PLoS One. 2015 Mar 13;10(3):e0117090. doi: 10.1371/journal.pone.0117090 (PMC4358884; doi:10.1371/journal.pone.0117090)
Supplement: S4 Information — (DOCX) [file pone.0117090.s004.docx]

**Online Supporting Information S4.** 27 candidate genes filtered by the second step of hybrid method and their maximum interaction score to reproduction-related gene

| **Ensembl ID** | **Gene symbol** | **Maximum interaction score to reproduction-related gene** |
| --- | --- | --- |
| ENSP00000364133 | TGFBR1 | 999 |
| ENSP00000380280 | FGFR1 | 999 |
| ENSP00000351905 | TGFBR2 | 999 |
| ENSP00000241416 | ACVR2A | 998 |
| ENSP00000266058 | SLIT1 | 997 |
| ENSP00000266646 | INHBE | 997 |
| ENSP00000309913 | TBX5 | 996 |
| ENSP00000263640 | ACVR1 | 994 |
| ENSP00000250448 | FOXA1 | 993 |
| ENSP00000245451 | BMP4 | 993 |
| ENSP00000168712 | FGF4 | 992 |
| ENSP00000254227 | NR0B2 | 989 |
| ENSP00000363708 | BMPR2 | 981 |
| ENSP00000364709 | F10 | 980 |
| ENSP00000277541 | NOTCH1 | 975 |
| ENSP00000379204 | BMP7 | 974 |
| ENSP00000295731 | IHH | 969 |
| ENSP00000256646 | NOTCH2 | 966 |
| ENSP00000264568 | BMPR1B | 944 |
| ENSP00000366534 | FOXH1 | 913 |
| ENSP00000355192 | CACNA1S | 907 |
| ENSP00000217086 | SALL4 | 894 |
| ENSP00000333203 | SERPINA5 | 844 |
| ENSP00000262238 | YY1 | 820 |
| ENSP00000234071 | PROC | 768 |
| ENSP00000363115 | FGR | 719 |
| ENSP00000249598 | GDF2 | 631 |
